# Assessment of the components of sarcopenia and quality of life perceived of individuals on hemodialysis

**DOI:** 10.1590/0034-7167-2022-0677

**Published:** 2023-12-04

**Authors:** Bianca Raquel Bianchi Celoto, Flávia Andréia Marin, Maria Claudia Bernardes Spexoto

**Affiliations:** I Universidade Federal da Grande Dourados. Dourados, Mato Grosso do Sul, Brazil

**Keywords:** Sarcopenia, Muscle Strength, Gait Speed, Quality of Life, Chronic Kidney Disease, Sarcopenia, Fuerza Muscular, Velocidad al Caminar, Calidad de Vida, Insuficiencia Renal Crónica, Sarcopenia, Força Muscular, Velocidade de Caminhada, Qualidade de Vida, Doença Renal Crônica

## Abstract

**Objectives:**

to evaluate the prevalence of sarcopenia in individuals aged 50 years or older on hemodialysis; to verify the association between sarcopenia and sociodemographic, clinical, anthropometric factors, components of sarcopenia and quality of life (QoL); and to correlate the components of sarcopenia with QoL.

**Methods:**

Participated 83 individuals on hemodialysis. Sarcopenia was established according to the current European consensus. Dynamometry to determine strength, calf circumference (CC) and appendicular skeletal muscle mass index (ASMMI) to obtain muscle mass and gait speed (GS) for physical performance. For QoL used the *WHOQOL-bref*.

**Results:**

the prevalence of sarcopenia was 32.6% (CC) and 18.1% (ASMMI). There was no association between sarcopenia and QoL. Both handgrip strength (r=0.25) and GS (r=0.36) showed a correlation with physical domain.

**Conclusions:**

sarcopenia was expressive, and the aspects of functionality determine the physical impairment in this population.

## RESUMO

**Objetivos:**

avaliar a prevalência de sarcopenia em indivíduos com 50 anos ou mais em hemodiálise, verificar a associação entre a sarcopenia e os fatores sociodemográficos, clínicos, antropométricos, componentes da sarcopenia e qualidade de vida (QV), e correlacionar os componentes da sarcopenia com a QV.

**Métodos:**

Participaram 83 indivíduos em hemodiálise. A sarcopenia foi estabelecida segundo consenso europeu vigente. A dinamometria para determinação da força, a circunferência da panturrilha (CP) e o índice de massa muscular esquelética apendicular (IMMEA) para a obtenção da massa muscular e a velocidade de caminhada (VC) para o desempenho físico. Para QV utilizou-se *WHOQOL-bref*.

**Resultados:**

a prevalência de sarcopenia foi de 32,6% (CP) e 18,1% (IMMEA). Não houve associação entre a sarcopenia e QV. Tanto a força de preensão manual (r=0,25) quanto a VC (r=0,36) apresentaram correlação com domínio físico.

**Conclusões:**

a sarcopenia foi expressiva e os aspectos da funcionalidade determinam o comprometimento físico nessa população.

## RESUMEN

**Objetivos:**

evaluar la prevalencia de sarcopenia en individuos de 50 años o más en hemodiálisis, verificar la asociación entre la sarcopenia y factores sociodemográficos, clínicos, antropométricos, componentes de la sarcopenia y la calidad de vida (CV), y para correlacionar los componentes de la sarcopenia con la CV.

**Métodos:**

Participaron 83 individuos en hemodiálisis. La sarcopenia se estableció de acuerdo con el consenso europeo vigente. Dinamometría para determinar la fuerza, la circunferencia de la pantorrilla (CP) y el índice de masa muscular esquelética apendicular (IMMEA) para obtener la masa muscular y la velocidad de la marcha (VM) para el rendimiento físico. Para CV el WHOQOL-bref.

**Resultados:**

la prevalencia de sarcopenia fue de 32,6% (CP) y 18,1% (IMMEA). No hubo asociación entre sarcopenia y CV. Tanto la fuerza de prensión manual (r=0,25) como la VM (r=0,36) se correlacionaron con el dominio físico.

**Conclusiones:**

la sarcopenia fue significativa y los aspectos de funcionalidad, determinan el deterioro físico en esta población.

## INTRODUCTION

Chronic kidney disease (CKD) is a public health problem, and its prevalence is increased in individuals over 65 years. Changes in metabolism and body composition, such as increased and redistributed body fat and reduced strength and muscle mass, are associated with the aging process and kidney disease per se, especially in individuals on hemodialysis (HD) treatment^(1-3)^.

Sarcopenia is diagnosed by the combination of low strength and low muscle mass, and its severity is determined by impairment of physical performance^(4)^ making it a concern among individuals with CKD, as studies have shown increased mortality risk and the likelihood of progression to end-stage renal disease (ESRD)^(5-8)^. The prevalences of sarcopenia found in the literature are discrepant, ranging from 4 to 68%, and vary significantly depending on the consensus adopted, the diagnostic methods used to assess muscle mass, and the cut-off points assigned for both low strength and low muscle mass^(1,9-10)^.

Among the studies that used the current criteria proposed by the European Working Group on Sarcopenia in Older People (EWGSOP2), Sánchez-Tocino et al.^(11)^ found 20% confirmed or severe sarcopenia. Yasar et al.^(12)^ found 29% sarcopenia, of these, 40% were on HD. Abdala et al.^(13)^ found a prevalence of sarcopenia and severe sarcopenia of 16% and 7%, respectively. Umakanthan et al.^(14)^ found 18% sarcopenia. And finally, Furtado et al.^(15)^, a Brazilian study, found 29.1% sarcopenia. All these studies were conducted with adult subjects and/or elderly people on HD. It is worth noting that Shu et al.^(9)^, in their systematic review and meta-analysis with 30 studies and 6,162 participating individuals, identified that the studies that considered only low muscle mass for the definition of sarcopenia was apparently higher than those that defined sarcopenia by the combination of low strength and low muscle mass.

The risk factors determining sarcopenia in CKD are diverse and may be related to different conditions, including CKD itself, treatment, and chronic low-grade inflammation. Other non-inflammatory causes are metabolic acidosis, insulin resistance, and vitamin D deficiency, which promote catabolism and decreased protein synthesis^(1)^. Risk factors, such as demographic, socioeconomic, and clinical aspects, may be related, but little is known about the prevalence of these factors in individuals with CKD on HD.

In addition to the high prevalence of sarcopenia in individuals with CKD, it is also associated with poorer quality of life (QoL), especially in those individuals on HD^(16)^. So, this is another aspect that deserves attention in these individuals because the evolution of therapies has provided increased survival and, therefore, both sarcopenia and QoL should be routinely assessed and monitored.

The QoL of this population may be associated with sarcopenia and its components (muscle strength, muscle mass, and physical performance), whose negative impacts include reduced mobility, increased risk and number of falls, loss of independence, increased hospitalizations, and increased risk of mortality^(17-19)^. In individuals with CKD on HD, QoL is assessed by means of general^(20-22)^ or specific^(23)^ instruments, validated with the purpose of measuring, in an individualized way, the situations that influence the individual’s well-being and his physical, functional, metabolic, social, and mental aspects^(24)^. It is known that QoL is a multidimensional concept that can be assessed by means of different strategies and/or tools, and most of them capture only QoL components. The use of tools such as the World Health Organization Quality of Life (WHOQOL), the WHOQOL-bref ^(22)^ helps to investigate the dimensions of QoL perceived by the individual. In this sense, assessing perceived QoL is important since the person’s perception can be crucial for the adherence and adoption of behaviors related to health and well-being.

Therefore, early investigation of the presence of sarcopenia and perceived QoL in individuals on HD, as well as the identification of its associated aspects, can contribute to improving the course of the disease and response to treatment. In the literature, studies have found an association between sarcopenia and QoL in elderly people^(20,25-27)^. Although much of this research was conducted with generic QoL instruments, these identified that sarcopenia declines QoL in this population. Above all, there are a limited number of studies that have investigated this relationship in the context of CKD^(20,28-29)^. Thus, knowing the prevalence of sarcopenia in this population and its associated factors, as well as the relationship between the components of sarcopenia and the domains of QoL, could promote the recognition of sarcopenia in the clinical setting and implement clinical-nutritional interventions that contribute to improvements in the course of the disease.

## OBJECTIVES

To assess the prevalence of sarcopenia in individuals aged 50 years or older with CKD on hemodialysis, to verify the association between sarcopenia and sociodemographic, clinical, and anthropometric factors, components of sarcopenia (muscle strength, skeletal muscle mass, and physical performance) and (QoL), and to correlate the components of sarcopenia with QoL.

## METHODS

### Ethical aspects

The study was approved by the Research Ethics Committee of the *Universidade Federal da Grande Dourados* (CEP-UFGD), being in accordance with the Declaration of Helsinki and Resolution No. 466/2012 of the Brazilian National Health Council. Written informed consent was obtained from all individuals involved in the study.

### Study design, setting, and period

This is a cross-sectional study, with non-probability sampling, conducted at *Clínica do Rim* - CENED (*Centro de Nefrologia de Dourados Ltda.*), a nephrology center located in the municipality of *Dourados*, in the state of *Mato Grosso do Sul*. The study was conducted from January 2021 to January 2022.

### Population, inclusion and exclusion criteria

The clinic attends an average of 170 patients per month, between private health plans and the public and free health plan - *Sistema Único de Saúde* [Brazilian Unified Health System] (SUS; https://www.gov.br/saude/pt-br/assuntos/saude-de-a-a-z/s/sus-estrutura-principios-e-como-funciona). In addition, it should be clarified that the clinic also serves the neighboring cities located in the *Grande Dourados* region and, to date, has 115 patients aged 50 years or older.

The inclusion criteria were individuals of both sexes, literate or not, aged 50 years or older, with a diagnosis of HD-dependent CKD. The age cutoff point of our population was chosen in order to identify more individuals with sarcopenia, since it is known that this muscle disease affects older adults and elderly people in a higher prevalence. We excluded bedridden patients and/or those who had any restrictions that made it impossible to perform the dynamometry and gait speed test, neurodegenerative diseases or severe psychiatric disorders confirmed in the patient’s medical record, and the indigenous population for ethical reasons.

### Study protocol and variables

#### Sociodemographic and clinical

The sociodemographic variables considered were age (complete years), sex (female/male), race - according to Lee et al.^(30)^ - Asian, African-American, and white or Hispanic), marital status (single, married, widowed, and divorced), work activity (absence/presence), and economic level - classified according to the Brazilian Economic Classification Criteria (ABEP) -, being level A (R$22,749.24), B (R$5,721.72 to R$10,788.56), C (R$1,894.95 to R$3,194.33), and D/E (R$862.41)^(31)^. Clinical variables were comorbidities (none, 1 to 2, 3 or more); functional capacity assessed by KPS (Karnofsky Performance Status) (0 to 100 points)^(32)^; metabolic stress - assessed by the 7-point Subjective Global Assessment tool (7-point SGA) -, which investigates diseases and comorbidities related to nutritional needs and classifies metabolic stress into normal, mild, moderate, and severe^(33-34)^; and treatment time (months), time to diagnosis of CKD (months) - obtained by the difference between the date of diagnosis and the date of Interview; and estimated glomerular filtration rate (EGFR; ml/min.).

Sociodemographic variables were obtained by personal interview at the time the patient was undergoing dialysis, and clinical variables were obtained directly by consulting the patient’s medical record.

#### Anthropometry

All evaluations were performed by trained researchers, without a pre-established order to avoid measurement and diagnosis biases, and each step was performed by only one professional. The anthropometric measurements used were weight (kg), height (m), and calf circumference (CC, cm), and were measured before the HD procedure for all subjects. Body weight was collected before and at the end of dialysis using a Toledo^®^ electronic scale, model 2003/26-2180, with maximum capacity of 250 kg, according to the clinic routine. Height was measured with the use of a stadiometer attached to the scale, according to the clinic routine; the patients remained with their backs to the rod, with arms extended along the body, with the palms of their hands facing it, barefoot, with heels together, and without adornments on the head.

Calf circumference was measured according to Lohman’s^(35)^ criteria, using an Essencial^®^ flexible and inelastic tape measure, graduated in cm, with a total length of 1.50 meters, positioned horizontally around the maximum calf circumference of the dominant leg. The individual remained seated with legs bent at a 90° angle.

The adductor pollicis muscle thickness (APMT, mm) was determined with the help of a Cescorf^®^ scientific adipometer. The measurement was performed with the individual seated, arm flexed at approximately 90°, with the forearm and hand resting on the knee. The individual was instructed to keep his hand relaxed. The pinched muscle is located at the vertex of an imaginary triangle formed by the extension of the thumb and index finger^(36)^. The procedure was performed in both hands, in triplicate, with a 1-minute interval between measurements, and the average between measurements was used.

#### Functional capacity evaluation

The functional capacity was assessed using the KPS index, with a score from 0 to 100, where 100 would correspond to “perfect health” and 0 “death”^(32)^.

The patient classification scale proposed by the instrument are: 100% (no signs or complaints, no evidence of disease), 90% (minimal signs and symptoms, carries out activities with effort), 80% (major signs and symptoms, performs their activities with effort), 70% (takes care of themselves, but not able to work), 60% (needs occasional assistance, but able to work), 50% (needs considerable assistance and frequent medical care), 40% (needs special medical care), 30% (extremely disabled, needs hospitalization but no imminent death), 20% (very ill, needs support), and 10% (dying, death imminent).

A higher score means that the patient is better able to carry out daily activities.

### Assessment and diagnosis of sarcopenia

#### Muscle strength

Handgrip strength (HGS, kg), measured prior to HD for all patients, was determined using a SAEHAN^®^ hand-held hydraulic dynamometer, model SH5001, with extremely accurate 0-90 Kg (0-200 lb) grip force measurement. The individual was first familiarized with the device and then examined while sitting with both arms bent with the elbow at 90°. The individual was instructed to hold the dynamometer and squeeze it at maximum force. The measurement was taken in triplicate in both hands, with a 1-minute interval between measurements, and the highest measurement was considered.

Low muscle strength was determined considering the cutoff points <32 kgf for men and <21 kgf for women^(37)^.

#### Skeletal muscle mass

In this study, low skeletal muscle mass was determined using two different methods: by obtaining the appendicular skeletal muscle index (ASMI); and by CC.

The Lee equation was used to estimate appendicular skeletal muscle mass (ASMM) = (0.244×body weight) + (7.8×height) + (6.6×sex) - (0.098×age) + (race-3.3), requiring the following information from the patient: weight in kg, height in meters, sex, age in years, and race. The value 0 was used for women and 1 for men. Values for race = -1.2 for Asians, 1.4 for African Americans, and 0 for Whites or Hispanics^(30)^. The ASMI was obtained from the result of the ASMM over the squared height of the individual, and the cutoff points adopted for low muscle mass were <9.1 kg/m^2^ for men and <6.6 kg/m^2^ for women, both determined at the 20th percentile of the sample distribution^(38-39)^.

Low muscle mass was also determined by CC, being ≤34 cm for men and ≤33 cm for women^(40)^.

#### Physical performance

Physical performance, measured before HD for all individuals, was obtained by gait speed (GS, meters/second). The individual was instructed to walk, at a habitual pace, a standard 4-meter (m) route three times, with an interval of 1 minute. The fastest route was considered. The criterion ≤0.8 m/s was used to determine low physical performance^(4)^.

#### Sarcopenia

After the evaluation of each of the components of sarcopenia, the responsible researcher made the diagnosis of sarcopenia separately in a second moment. The diagnosis and prevalence of sarcopenia was made according to the algorithm proposed by the EWGSOP2^(4)^. It should be clarified that all individuals were considered clinically suspect in the initial screening for this research, considering that individuals with CKD on hemodialysis treatment, by themselves, already have signs suggestive of sarcopenia. This criterion was adopted instead of using the SARC-F instrument.

Patients were categorized into 1. non-sarcopenia, 2. probable sarcopenia, when satisfying the criterion of low muscle strength, 3. confirmed sarcopenia, when in addition to low muscle strength, they also presented low muscle quantity, and 4. severe sarcopenia, when they presented the two previous criteria associated with low physical performance.

#### Quality of life (QoL)

Quality of life was assessed using the brief version of the World Health Organization Quality of Life instrument, the WHOQOL-bref^(41)^, developed and proposed by the WHOQOL Group in 1998, which derives from the full version, the WHOQOL-100. The instrument has been translated into Portuguese and validated for the Brazilian population^(42)^. It is composed of 26 items and is structured by a Likert-type response scale of five points, in which the individual’s perception may vary as to intensity (not at all to an extreme amount), capacity (not at all to completely), frequency (never to always), and evaluation (very dissatisfied to very satisfied; very poor to very good). Each domain has a total score ranging from 0 to 100 points; and the higher the score, the better the QoL. Twenty-four items are divided into four factors (domains) called: Physical Health domain (PH) (item 3 = pain; 4 = medication; 10 = energy and fatigue; 15 = mobility; 16 = sleep and rest; 17 = activities of daily living; 18 = work capacity), Psychological Health domain (PSH) (item 5 = positive feeling; 6 = spirituality, religion, and personal beliefs; 7 = thinking, learning, memory, and concentration; 11 = bodily image and appearance; 19 = self-esteem; 26 = negative feeling), Social Relationships (SR) (item 20 = personal relationships; 21 = sexual activity; 22 = social support), and Environment quality of life (EQL) (item 8 = freedom, physical safety, and security; 9 = physical environment; 12 = financial resource; 13 = opportunities for acquiring new information and skills; 14 = participation in and opportunities for recreation/leisure activities; 23 = home environment; 24 = health and social care: accessibility and quality; 25 = transportation). Two other items assess general quality of life (item 1) and general health (item 2)^(41)^.

The instrument was answered based on the last two weeks of the individual’s life. Although it is a self-assessment and self-explanatory instrument, due to health or literacy conditions it was read and filled-out by the interviewers.

### Data Analysis

For the statistical analyses, the individuals identified with confirmed and severe sarcopenia composed the same group (sarcopenia).

Initially, the normality of the variables was tested using the Shapiro Wilk test (p>0.05). Data were expressed as mean and standard deviation for continuous variables and percentages for categorical variables. Continuous variables were also presented as medians when there was a high standard deviation. Pearson’s chi-square (χ^2^) test was used for associations between sarcopenia, as determined by CC or ASMI, and sociodemographic, clinical, anthropometric, and sarcopenia component variables. Analysis of variance (ANOVA) was used for comparison of means between sarcopenia groups. Multiple comparisons were performed using Tukey’s test.

Welch’s correction was used in case of violation of the homoscedasticity assumption, and in this condition, the Games-Howell post-test was adopted. To estimate the correlation between the components of sarcopenia and quality of life, Pearson’s correlation coefficient (r) was used, considering r = 0 null correlation, r > 0 and ≤ 0.30 weak, r > 0.30 and ≤ 0.50 moderate, r > 0.5 and ≤ 0.70 strong, and r > 0.70 very strong. A significance level of 5% (p<0.05) was adopted for all analyses.

## RESULTS

**Figure 1:**
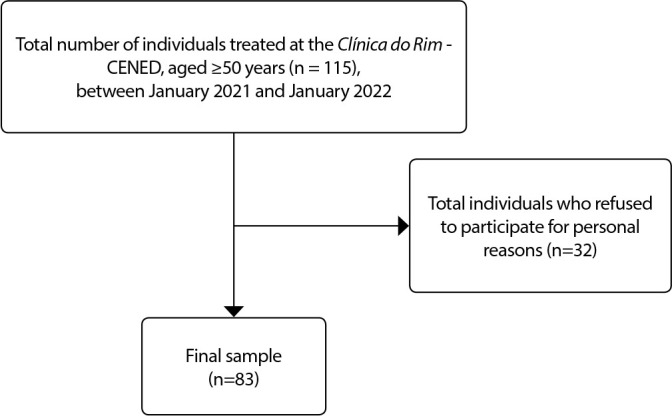
Sample Flowchart

This study was composed of 83 patients with a mean age of 61.8 ± 8.3 years. Figure 1 presents the sample flowchart.

*CENED: Centro de Nefrologia de Dourados “Dourados Nephrology Center”.*

There was a predominance of males (72.3%), elderly people (53.0%), whites and Hispanics (53.0%), married (59.0%), with no work activity (89.2%), belonging to the economic level C (51.8%), with 1 to 2 comorbidities (73.5%), no metabolic stress (80.7%), and with excellent functional capacity (53.0%). Regarding sarcopenia components, most presented low muscle strength (67.5%), adequate muscle mass both by CC (55.4%) and ASMI (79.5%), and adequate GS (71.1%). In the QoL investigation, PH presented the lowest score (61.1±16.5; p<0.001). The median duration of HD in the study population was 26 months (8-49 months).; the median time to diagnosis of CKD was 30 months (11-45 months); and the mean eGFR was 7.9±3.9 ml / min. The median time of HD in the study population was 26 months (p25: 8 months; p75: 49 months). Regarding anthropometry, the subjects had a mean weight of 70.2±13.9 kg, height of 1.66±0.1 m, BMI of 25.6±4.5 kg/m^2^, APMT of 9.6±3.7 mm and 9.4±4.1 in the right and left hands, respectively. (Table 1).

**Table 1:** Sociodemographic, clinical, anthropometric, sarcopenia components, and quality of life characteristics of individuals (N=83)

Variables	n	%
	M±SD	
*Sociodemographic*		
Age (years)	61.8±8.3	
Age group		
Adult	39	47.0
Elder	44	53.0
Sex		
Male	60	72.3
Female	23	27.7
Race		
White/Hispanic	44	53.0
Black/African-American	38	45.8
Asian	1	1.2
Marital status		
Married	49	59.0
Single	8	9.6
Widowed	16	19.3
Divorced	10	12.0
Work activity		
No	74	89.2
Yes	9	10.8
Economic level		
A	-	-
B	20	24.1
C	43	51.8
D and E	20	24.1
*Clinical*		
Comorbidities		
None	6	7.2
1 to 2	61	73.5
3 or more	16	19.3
Functional capacity (KPS)		
No signs or complaints; no evidence of disease (100%)	44	53.0
Stress		
None	67	80.7
Low	7	8.4
Moderate	7	8.4
Elevated	2	2.4
Time in treatment (months)	34.0±32.8	
*Anthropometry*		
Weight (kg)	70.2±13.9	
Height (m)	1.66±0.08	
BMI (kg/m^2^)	25.6±4.5	
APMT R (mm)	9.6±3.7	
APMT L (mm)	9.4±4.1	
*Sarcopenia components*		
Calf circumference		
CC (cm)	34.7±3.7	
Adequate	46	55.4
Low muscle mass	37	44.6
ASMI		
ASMI (kg/m^2^)	9.4±1.7	
Adequate	66	79.5
Low muscle mass	17	20.5
Handgrip strength		
HGS (kgf)	26.6±10.3	
Adequate	27	32.5
Low muscle strength	56	67.5
Gait speed		
GS (m/s)	0.91±0.26	
Adequate	59	71.1
Low gait speed	24	28.9
Quality of life (QoL)		
Physical Health (PH)	61.1±16.5	
Psychological Health (PSH)	79.4±17.8	
Social Relationships (SR)	75.6±24.0	
Environment quality of life (EQL)	79.9±15.2	
Global QoV	67.6±20.9	

*KPS: Karnofsky Performance Status; BMI: Body mass index; APMT: adductor pollicis muscle thickness; R: right; L: left; CC: calf circumference; ASMI: appendicular skeletal muscle index; HGS: handgrip strength; GS: gait speed; m/s: meters per second; QoL: quality of life; M: Mean; SD: standard deviation.*

Low skeletal muscle mass was determined by CC and ASMI. Using the CC, there was a predominance of individuals with probable sarcopenia (34.9%), while confirmed and severe sarcopenia totaled 32.6%. Using the ASMI, probable sarcopenia was also predominant (49.4%), and the total number of patients with sarcopenia (confirmed and severe) was 18.1% (Table 2).

**Table 2:** Prevalence of sarcopenia with skeletal muscle mass estimated by calf circumference (CC) and appendicular skeletal muscle index (ASMI)

	Skeletal muscle mass	
**Sarcopenia**	**CC**	**ASMI**
	**n(%)**	**n(%)**
4-Category Sarcopenia		
Non-sarcopenia	27(32.5)	27(32.5)
Probable sarcopenia	29(34.9)	41(49.4)
Confirmed sarcopenia	17(20.5)	9(10.8)
Severe sarcopenia	10(12.1)	6(7.3)
3-Category Sarcopenia		
Non-sarcopenia	27(32.5)	27(32.5)
Probable sarcopenia	29(34.9)	41(49.4)
Sarcopenia (confirmed and severe)	27(32.6)	15(18.1)

*CC: calf circumference; ASMI: appendicular skeletal muscle index*

When sarcopenia was determined by CC measurement, associations were observed with weight (p<0.001), height (p=0.004), BMI (p<0.001), ASMI (<0.001), HGS (<0.001), and GS (p=0.002). When determined by ASMI, there was an association between sarcopenia and the variables weight (p<0.001), height (p=0.006), BMI (p<0.001), CC (<0.001), HGS (<0.001), and GS (p=0.002) (Table 3).

**Table 3:** Distribution of individuals according to sarcopenia classification and association with sociodemographic, clinical, anthropometric characteristics, sarcopenia components, and quality of life

	Sarcopenia by CC				Sarcopenia by ASMI			
Variables	Non-sarcopenia	Probable sarcopenia	Sarcopenia	*p* value^*^	Non-sarcopenia	Probable sarcopenia	Sarcopenia	*p* value^*^
	n(%)	n(%)	n(%)		n(%)	n(%)	n(%)	
*Sociodemographic*								
Age (years)	59.4±7.9 (56.7-62.2)	62.6±6.9 (60.1-65.6)	63.4±9.4 (59.8-66.9)	0.172	59.4±7.9 (56.2-62.8)	61.4±6.5 (59.4-63.4)	67.4±10.5 (61.0-73.9)	0.060
Age group				0.122				0.111
Adult	17(43.6)	12(30.8)	10(25.6)		17(43.6)	17(43.6)	5(12.8)	
Elder	10(22.7)	17(38.6)	17(38.6)		10(22.7)	24(54.5)	10(22.7)	
Sex				0.059				0.062
Male	24(40.0)	18(30.0)	18(30.0)		24(40.0)	26(43.3)	10(16.7)	
Female	3(13.0)	11(47.8)	9(39.1)		3(13.0)	15(65.2)	5(21.7)	
Race				0.660				0.401
White/Hispanic	14(31.8)	14(31.8)	16(36.4)		14(31.8)	19(43.2)	11(25.0)	
Black/African-American	13(34.2)	14(36.8)	11(28.9)		13(34.2)	21(55.3)	4(10.5)	
Asian	-	1(100.0)	-		-	1(100.0)	-	
Marital status				0.548				0.575
Married	15(30.6)	19(38.8)	15(30.6)		15(30.6)	26(53.1)	8(16.3)	
Single	4(50.0)	2(25.0)	2(25.0)		4(50.0)	4(50.0)	-	
Widowed	4(25.0)	7(43.8)	5(31.3)		4(25.0)	7(43.8)	5(31.3)	
Divorced	4(40.0)	1(10.0)	5(50.0)		4(40.0)	4(40.0)	2(20.0)	
Work activity				0.678				0.546
No	25(33.8)	26(35.1)	23(31.1)		25(33.8)	35(47.3)	14(18.9)	
Yes	2(22.2)	3(33.3)	4(44.4)		2(22.2)	6(66.7)	1(11.1)	
Economic level				0.907				0.375
A	-	-	-		-	-	-	
B	7(35.0)	7(35.0)	6(30.0)		7(35.0)	7(35.0)	6(30.0)	
C	12(27.9)	16(37.2)	15(349)		12(27.9)	24(55.8)	7(16.3)	
D and E	8(40.0)	6(30.0)	6(30.0)		8(40.0)	10(50.0)	2(10.0)	
*Clinical*								
Comorbidities				0.238				0.390
None	3(50.0)	3(50.0)	-		3(50.0)	2(33.3)	1(16.7)	
1 to 2	20(32.8)	18(29.5)	23(37.7)		20(32.8)	28(45.9)	13(21.3)	
3 or more	4(25.0)	8(50.0)	4(25.0)		4(25.0)	11(68.8)	1(6.3)	
Metabolic stress				0.291				0.693
None	22(32.8)	26(38.8)	19(28.4)		22(32.8)	33(49.3)	12(17.9)	
Low	3(42.9)	2(28.6)	2(28.6)		3(42.9)	4(57.1)	-	
Moderate	2(28.6)	1(14.3)	4(57.1)		2(28.6)	3(42.9)	2(28.6)	
Elevated	-	-	2(100.0)		-	1(50.0)	1(50.0)	
Disease information								
Treatment time (months)	33.5±27.8 (23.9-44.5)	35.6±35.3 (24.5-47.6)	36.9±37.6 (22.1-53.6)	0.930	33.5±27.8 (23.7-44.7)	38.0±37.7 (27.2-50.5)	31.4±32.8 (16.4-49.2)	0.789
Time of kidney disease (months)	34.5±27.2 (11.0-53.0)	38.4±34.8 (13.0-50.5)	33.3±33.0 (8.0-43.0)	0.667	34.5±7.5 (11.0-53.0)	37.6±33.7 (15.5-44.0)	31.4±34.4 (6.0-58.0)	0.641
TFGe (ml/min)	7.5±3.3 (4.9-6.9)	7.4±2.7 (5.2-9.5)	8.8±5.2 (5.8-9.3)	0.486	7.5±3.3 (4.9-9.4)	7.9±4.3 (5.3-9.5)	8.3±3.7 (6.2-9.3)	0.611
*Anthropometry*								
Weight (kg)	75.4±15.8 (70.0-81.2)^a^	74.1±8.9 (70.5-77.7)^a^	58.9±7.8 (55.9-61.9)^b^	**<0.001**	75.4±15.8 (69.8-81.6)^a^	70.3±10.2 (66.8-73.5)^a^	57.1±8.1 (53.1-61.7)^b^	**<0.001**
Height (m)	1.70±0.08 (1.67-1.73)^a^	1.65±0.08 (1.62-1.68)^a.b^	1.62±0.08 (1.59-1.65)^b^	0.004	1.70±0.08 (1.66-1.73)^a^	1.63±0.08(1.60-1.66)^b^	1.65±0.07(1.61-1.69)^a.b^	0.006
BMI (kg/m^2^)	26.2±5.4 (24.3-28.4)^a^	27.4±3.1 9 (26.1-28.7)^a^	22.3±2.3 (21.5-23.2)^b^	**<0.001**	26.2±5.4 (24.5-28.2)^a^	26.4±3.1 (25.3-27.5)^a^	20.9±2.0 (19.8-22.1)^b^	**<0.001**
APMT R (mm)	9.8±3.3 (8.6-11.2)	9.7±3.8 (8.4-11.2)	9.4±4.2 (7.7-11.0)	0.910	9.8±3.3 (8.7-11.0)	9.3±3.7 (8.2-10.5)	10.3±4.7 (7.8-13.0)	0.699
APMT L (mm)	10.3±5.9 (8.7-12.5)	9.4±2.7 (8.4-10.5)	8.5±3.1 (7.2-9.6)	0.290	10.3±5.9 (8.5-12.4)	9.1±2.7 (8.3-10.0)	8.6±3.3 96.9-10.3)	0.483
*Sarcopenia components*								
Calf circumference				**<0.001**				0.010
Adequate	17 (37.0)	29 (63.0)	-		17(37.0)	26(56.5)	3(6.5)	
Low muscle mass	10 (27.0)	-	27 (73.0)		10(27.0)	15(40.5)	12(32.4)	
CC (cm)	36.0±3.8 (34.5-37.5)^a^	37.0±2.0 (36.1-37.7)^a^	31.0±2.1 (30.2-31.8)^b^	**<0.001**	36.0±3.8 (34.5-37.5)^a^	35.1±3.0 (34.2-36.1)^a^	31.0±3.5 (29.1-33.0)^b^	**<0.001**
ASMI				**0.001**				**<0.001**
Adequate	25(37.9)	26(39.4)	15(22.7)		25(37.9)	41(62.1)	-	
Low muscle mass	2(11.8)	3(17.6)	12(70.6)		2(11.8)	-	15(88.2)	
ASMI (kg/m2)	10.1±1.8 (9.4-10.8) ^a^	9.8±1.4 (9.2-10.3) ^a^	8.4±1.4 (7.9-9.0) ^b^	**<0.001**	10.1±1.8 (9.4-10.8)^a^	9.6±1.4 (9.1-10.0)^a^	7.9±1.1 (7.2-8.5)^b^	**<0.001**
Handgrip strength				**<0.001**				**<0.001**
Adequate	27(100.0)	-	-		27(100.0)	-	-	
Low muscle strength	-	29(51.8)	27(48.2)		-	41(73.2)	15(26.8)	
HGS (kgf)	38.5±7.0 (35.7-41.2)^a^	20.8±5.7 (18.6-22.9)^b^	21.1±6.2 (18.7-23.5)^b^	**<0.001**	38.5±7.0 (35.7-41.2)^a^	21.6±5.7 (19.8-23.4)^b^	19.3±6.2 (15.8-22.7)^b^	**<0.001**
Gait speed				0.141				0.132
Adequate	23(39.0)	19(32.2)	17(28.8)		23(39.0)	27(45.8)	9(15.3)	
Low gait speed	4(16.7)	10(41.7)	10(41.7)		4(16.7)	14(58.3)	6(25.0)	
GS (m/s)	1.05±0.23 (0.95-1.14)^a^	0.86±0.25 (0.77-0.96)^b^	0.81±0.24 (0.72-0.91)^b^	0.002	1.05±0.24 (0.95-1.14)^a^	0.85±0.23 (0.78-0.92)^b^	0.81±0.30 (0.64-0.97)^b^	0.002
Quality of life (QoL)								
Physical Health (PH)	65.7±14.0 (60.2-71.3)	60.5±17.5 (53.8-67.1)	57.3±17.2 (50.4-64.1)	0.165	65.7±14.0 (60.2-71.3)	60.0±16.6 (54.7-65.3)	55.9±19.3 (45.3-66.6)	0.153
Psychological Health (PSH)	83.3±16.4 (76.3-89.7)	77.1±19.3 (68.7-84.8)	80.7±15.3 (73.8-87.0)	0.458	83.3±16.4 (77.2-89.9)	79.7±18.5 (73.4-85.8)	76.5±14.6 (69.1-84.0)	0.399
Social Relationships (SR)	78.1±24.5 (68.1-87.5)	73.7±26.6 (62.9-83.4)	76.0±23.1 (66.2-85.0)	0.827	78.1±24.5 (69.2-85.9)	77.5±24.6 (67.9-85.9)	67.8±24.6 (54.2-81.3)	0.411
Environment quality of life (EQL)	84.7±13.1 (78.9-90.1)	77.5±15.7 (71.4-83.3)	76.5±16.3 (70.1-82.8)	0.086	84.7±13.1 (79.3-89.7)	77.5±15.4 (72.5-82.9)	75.7±17.2 (66.2-85.60	0.092
Global QoV	65.7±21.0 (57.8-73.8)	71.1±22.0 (62.4-80.0)	66.0±20.0 (57.5-74.8)	0.600	65.7±21.0 (58.4-72.9)	70.6±22.1 (63.6-77.8)	63.4±17.3 (54.5-72.0)	0.444

CC: calf circumference; ASMI: appendicular skeletal muscle index; BMI: Body mass index; APMT: adductor pollicis muscle thickness. EGFR: estimated glomerular filtration rate.

* Pearson's chi-square (categorical) or ANOVA (means; continuous). Matching letters: statistical similarity.

In both muscle mass assessment methods, weight and BMI measurements were lower (p<0.001) in patients with sarcopenia compared to the non-sarcopenia and probable sarcopenia groups. Regardless of the method of muscle mass assessment, the sarcopenia group differed from the others in CC and ASMI, being lower in the sarcopenia group. In contrast, in the HGS and GS components, the differences observed were between the sarcopenia and non-sarcopenia groups (Table 3).

There was no association between sarcopenia and global QoL as well as the other domains evaluated (Table 3).

Positive correlations were observed between HGS and PH (r=0.25; weak), and between GS and PH (r=0.36; moderate), and GS and EQL (0.22; weak) (Table 4).

**Table 4:** Correlation coefficient of sarcopenia components (muscle strength, skeletal muscle mass, and physical performance) and quality of life

	HGS	CC	ASMI	GS	PH	PSH	SR	EQL	Global QoV
HGS	1	0.26^*^	0.46^**^	0.51^**^	0.25^*^	0.18(0.118)	0.18(0.115)	0.19(0.096)	-0.002(0.984)
CC		1	0.53^**^	0.01(0.918)	0.06(0.624)	0.05(0.661)	0.03(0.789)	0.10(0.384)	0.08(0.470)
ASMI			1	0.06(0.579)	0.11(0.343)	0.15(0.194)	0.06(0.565)	0.14(0.219)	0.10(0.383)
GS				1	0.36^**^	0.18(0.116)	0.13(0.242)	0.22^*^	0.12(0.297)
PH					1	0.72^**^	0.40^**^	0.60^**^	0.36^**^
PSH						1	0.43^**^	0.69^**^	0.41^**^
SR							1	0.38^**^	0.15(0.172)
EQL								1	0.42^**^
Global QoV									1

* *significance at the 0.05 level;*

* significance at the 0.001 level; HGS: handgrip strength; CC: calf circumference; ASMI: appendicular skeletal muscle index; GS: gait speed; PH: physical health; PSH: psychological health; SR: social relationships; EQL: environment quality of life; QoL: quality of life.

## DISCUSSION

This study showed that there was no association between sarcopenia and quality of life. In contrast, sarcopenia showed association with conventional anthropometric measurements and with all the components used for its diagnosis. When the correlation of each of the components of sarcopenia (HGS, CC, ASMI, and GS) with global QoL and its domains was evaluated, we found significance between HGS and PH, between GS and PH, and finally, between GS and EQL. Giglio et al.^(2)^ also found that muscle strength was associated with worse scores in the QoL domains, since weakness is part of the aging process and compromises physical performance and basic activities of daily living^(2,43)^.

What also emerges from the present study is that physical functioning plays an extremely important role in QoL. The literature points out that the main drivers of QoL in elderly people are usually having energy, the absence of pain, the ability to perform basic and instrumental activities of daily living, and being able to move^(3,17-18)^. Therefore, any of these “threats” can negatively impact the QoL of elderly people, especially in the presence of kidney disease in HD treatment. This is observable in our study when the components of physical function, HGS and GS, were associated with PH.

Another relevant finding was that the measures used to estimate muscle mass, CC and ASMI, showed no correlation with overall QoL nor with the domains evaluated. This fact can be explained by the influence that HGS and GS components exert on QoL. GS, by itself, besides being an important indicator of old age, is an outcome measure, i.e., the elderly person who is slow is very likely to already be debilitated. It is worth noting that our study is mostly composed of elderly people with no work activity, i.e., possibly retired, and consequently, more sedentary/inactive, being one of the risk factors for reduced physical function in the elderly^(44)^.

Furthermore, we identified that sarcopenia (confirmed and severe) was worrisome among individuals with CKD, on hemodialysis, and aged 50 years or more, and the prevalence was almost twice as high when low muscle mass was determined by CC. Although in the present study muscle mass was assessed by the CC and predictive equation methods for obtaining the ASMI, our prevalences were similar to those found by Sánchez-Tocino, who found 20%^(11)^; Yasar et al., who found 29%^(12)^; Abdala et al.^(13)^, who found 16% confirmed sarcopenia and 7% severe sarcopenia; Umakanthan et al.^(14)^, who found 18%; and Furtado et al.^(15)^, who found 11.5%.

The number of individuals with low muscle strength in the present study draws attention, contrasting with Lee et al.^(45)^ who used cutoff values for low HGS of 29.5 kg for men and 16.8 kg for women, and found 25.2% of low HGS. We believe that this occurred due to the cutoff points that were chosen (<32/21 kg) to determine low muscle strength, because the higher the cutoff points are, the more individuals have the chance of having their muscle mass evaluated. It is important to highlight that, the individuals, when being evaluated by the algorithm flow determined by EWGSOP2, are still diluted between the confirmed sarcopenia and severe sarcopenia categories.

We found no association between sarcopenia and perceived QoL. Therefore, we speculate two possible explanations for this fact. The first is that our study is composed of a sample of ‘younger’ elderly people, on average 61.8 years, and, consequently, with less impairment in functionality. Second, the instrument we adopted for the assessment of QoL is not specific for CKD, which may have interfered in the capture of this construct. However, it is known that sarcopenia is associated with an obvious decline in QoL in elderly people^(2,20)^, especially in PH^(6,28)^, and this indicates the importance of preventive and interventional management strategies for the early diagnosis of sarcopenia in these individuals. These assessments are important for dialysis service providers to understand the needs and concerns of important segments of this population, and to allocate resources and define reforms and initiatives to improve clinical-nutritional care.

### Strengths and limitations of the study

This study has strengths and limitations that should be highlighted. As strengths, it has a sample composed of individuals from different cities located in the region of *Grande Dourados* and this may represent the macro-region of *Dourados* in *Mato Grosso do Sul* (MS), even if performed in a single center for dialysis treatment. Furthermore, it is the first Brazilian study carried out in the state of MS, central-western region of Brazil, with individuals with CKD on HD.

As limitations we point out, first, that because it is a cross-sectional study it is not possible to establish causal relationships. Second, the methods used to assess the amount of muscle mass are not robust and not recommended by the European consensus, but the study used accessible methods that can be easily and alternatively replicated both in future research and in clinical practice. Finally, the study used a generic instrument to assess quality of life rather than one specific to CKD and this may have resulted in insensitivity to any conditions related to kidney disease; however, generic instruments are widely used in disease settings and allow comparisons with other population groups.

### Contributions to the field of nursing, nutrition, health, or public health

The results of this study suggest that the screening of muscle strength, using dynamometry, and muscle mass, even if in possession of more accessible methods for the determination of the diagnosis of sarcopenia, can contribute to a better quality of life of this population, especially in the physical aspect. Therefore, it must be incorporated into the clinical routine - both in the initial evaluation and in the monitoring of these individuals. It is also expected that this study will contribute to the implementation of the evaluation of muscle health as a fundamental part of clinical and nutritional treatment, seeking dietary interventions and strategic approaches to prevent sarcopenia as well as the worsening of physical health of patients on hemodialysis, thus minimizing the possible unfavorable outcomes.

## CONCLUSIONS

A probable sarcopenia was the status with the highest prevalence, and the physical domain regarding the evaluation of perceived QoL was the most affected in the population of the present study. We also identified that individuals with sarcopenia had lower weight, body mass index, CC, and ASMI, compared to individuals diagnosed as non-sarcopenia and probable sarcopenia. The aspects of functionality, HGS and CC, determine the physical impairment in this population. We also highlight that the finding of prevalence of sarcopenia using the CC method was almost twice as high compared to the ASMI.
